# Development of a tool to recognize small airways dysfunction in asthma (SADT)

**DOI:** 10.1186/s12955-014-0155-7

**Published:** 2014-11-22

**Authors:** Lieke Schiphof-Godart, Erica van der Wiel, Nick HT ten Hacken, Maarten van den Berge, Dirkje S Postma, Thys van der Molen

**Affiliations:** Department of General Practice, University of Groningen, University Medical Center Groningen, Groningen, the Netherlands; University of Groningen, University Medical Center Groningen, Groningen Research Institute for Asthma and COPD (GRIAC), A. Deusinglaan 1, 9700 AV, PO Box 196, Groningen, HPC FA 21 The Netherlands; Department of Pulmonary Medicine and Tuberculosis, University of Groningen, University Medical Center Groningen, Groningen, the Netherlands

**Keywords:** Small airways dysfunction, Asthma, Questionnaire

## Abstract

**Background:**

Small airways dysfunction (SAD) contributes to the clinical expression of asthma. The identification of patients who suffer from SAD is important from a clinical perspective, as targeted therapy may improve patients’ well-being and treatment efficacy.

**Aims:**

We aimed to realize the first step in the development of a simple small airways dysfunction tool (SADT) that may help to identify asthma patients having SAD.

**Methods:**

Asthma patients with and without SAD were interviewed. Patients were selected to participate in this study based on FEF_50%_ and R5-R20 values from spirometry and impulse oscillometry respectively.

**Results:**

Ten in depth interviews and two focus groups revealed that patients with and without SAD perceived differences in symptoms and signs, habits and health related issues. For example, patients with SAD reported to wheeze easily, were unable to breathe in deeply, mentioned more symptoms related to bronchial hyperresponsiveness, experienced more pronounced exercise-induced symptoms and more frequently had allergic respiratory symptoms after exposure to cats and birds. Based on these differences, 63 items were retained to be further explored for the SADT.

**Conclusions:**

The first step of the development of the SADT tool shows that there are relevant differences in signs and respiratory symptoms between asthma patients with and without SAD. The next step is to test and validate all items in order to retain the most relevant items to create a short and simple tool, which should be useful to identify asthma patients with SAD in clinical practice.

**Electronic supplementary material:**

The online version of this article (doi:10.1186/s12955-014-0155-7) contains supplementary material, which is available to authorized users.

## Introduction

Asthma is one of the most common chronic diseases in people of all ages in developed countries [1]. Frequently reported symptoms are breathlessness, chest tightness, wheeze, cough, limitation of physical activity, and nocturnal awakening. Large airways obstruction due to inflammation and remodeling was traditionally thought to be the origin of these symptoms. However, there is growing consensus that the small airways are also affected, and play a role in the clinical expression of asthma [2,3]. A recent systematic review showed that small airways dysfunction (SAD) is associated with worse asthma control, a higher number of exacerbations, the presence of nocturnal asthma, more severe bronchial hyperresponsiveness (BHR) and exercise-induced asthma [4]. Moreover, clinical studies have shown that small particle treatment with inhaled corticosteroids reduces the number of exacerbations and improves asthma control [5-7]. Thus, it has become increasingly important to identify those asthma patients in whom SAD is present. Several tests are available to assess SAD in patients with asthma, like the forced expiratory flow rates at 50 or at 25 to 75% of the forced vital capacity (FEF_50%_ or FEF_25–75%_) which can easily be assessed with spirometry [8-10]. This FEF is closely related to air trapping on an expiratory CT-scan [11,12]. In addition, impulse oscillometry (IOS) has been used as an easy tool to measure the resistance of the small and large airways [10].

Another method that could help to assess the presence of SAD in asthma patients is by identifying symptoms associated with SAD. These could then be used in a questionnaire to assess both the probability of SAD and the burden of symptoms associated with SAD. So far, it has not been studied whether small or large airways obstruction in asthma generates different symptoms. This may well be the case, since small airways have a smaller lumen size than large airways and lack cartilage. Therefore, smooth muscle contraction may lead to a collapse of the small airways, contributing to air trapping and the perception of chest tightness [13]. Additionally, there is a difference in vagal innervation between the large airways and deeper lung structures, including the small airways [14,15]. Finally, not all environmental stimuli are able to reach the small airways. This depends on the particle size, aerodynamic properties and local airway flow characteristics. For instance, cat allergen may reach the peripheral airways, whereas most pollen will never do so because of their large particle size [16,17].

Thus, this study aims to determine which self-reported differences in symptoms might potentially differentiate between asthma patients with SAD and without SAD. In the future, these items might be used to create a tool to recognize SAD in asthma in daily clinical practice, the SADT.

## Methods

### Selection of study population

**Figure 1:**
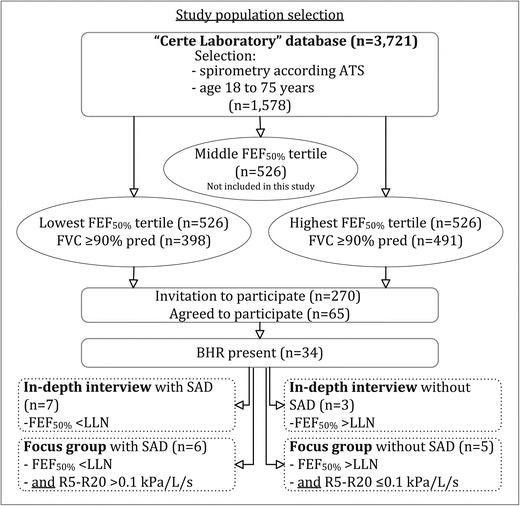
**Flowchart of the study with the selection of the study population.** ATS: American Thoracic Society, BHR: Bronchial hyperresponsiveness, FEV_1_= Forced expiratory flow in one second, FVC= forced vital capacity, FEF_50%_= forced expiratory flow at 50% of the FVC, LLN: lower limit of normal, R5-R20: Difference between the resistance at 5 Hz and 20 Hz.

The participants were selected out of a database of patients attending the Primary Care asthma/COPD service of “Certe Laboratory” (The Netherlands) [18]. This database contains 3,721 patients with a doctor’s diagnosis of asthma. We selected asthma patients aged between 18 and 75 years with spirometry according to the American Thoracic Society (ATS) criteria available (Figure 1). This group (n= 1,578) was divided into three groups (tertiles) based on their post-bronchodilator FEF_50%_ percent predicted, in order to select patient populations with and without probable SAD. The 33% patients (n= 526) with the lowest FEF_50%_ represented the group of asthma patients with probable SAD and the 33% patients (n= 526) with the highest FEF_50%_ the group (probably) without SAD. Since a restrictive lung function may lead to low FEF_50%_ predicted values and the false interpretation of existing SAD, we decided to exclude patients with an FVC<90% predicted, leaving 398 patients in the group with SAD and 491 patients in the group without SAD. Characteristics of the source population are shown in Table 1.

**Table 1:** **Characteristics of source population divided into the lowest and highest FEF**_**50%**_ **tertile**

	**Lowest FEF**_**50%**_ **tertile**		**Highest FEF**_**50%**_ **tertile**	
	**(n= 398)**		**(N= 491)**	
Age (years)	54	(12)	48	(13)
Gender (%female)	72		62	
BMI (kg/m^2^)	28	(5)	29	(6)
Smoking (%current/ex/never )	26/37/37		15/42/43	
ICS (%yes)	53		50	
FEV_1_ (%predicted)	89	(9.8)	109	(12)
FEV_1_/FVC (%)	70	(5.7)	83	(4.3)
FEF_50%_ (%predicted)	51	(9.9)	104	(17)
ACQ total score	1.3	(0.9)	1.2	(0.9)

Data presented as mean (SD) or percentage.

BMI= body mass index, ICS= inhaled corticosteroids, FEV_1_= Forced expiratory flow in one second, FVC= forced vital capacity, FEF_50%_= forced expiratory flow at 50% of the FVC, ACQ= Asthma Control Questionnaire.

General practitioners were contacted and asked for permission to contact their patients eligible for this study. Patient recruitment continued until saturation with respect to content was reached. Finally, 120 patients from the lowest tertile and 150 patients from the highest tertile were invited to participate in this study and a total of 65 patients accepted to participate. They completed the asthma control questionnaire (ACQ), the Clinical COPD Questionnaire (CCQ) and the Bronchial Hyperresponsiveness Questionnaire (BHQ), and performed measurements of spirometry, impulse oscillometry and a methacholine provocation test (methods of the measurements are described in the Additional file 1). Four patients were unable to perform spirometry and were excluded. Patients with BHR (n= 34), i.e. a provocative concentration causing a 20% fall in FEV_1_ (PC_20_) methacholine bromide ≤39.3 mg/ml, were included for further analysis.

### In-depth interviews and focus groups

First, explorative interviews were performed aiming to collect topics for the focus group interviews. For these in-depth interviews, 7 patients with and 3 without SAD were selected, based on current FEF_50%_ values lower or higher than the lower limit of normal (LLN). One patient from the highest tertile FEF_50%_ had an FEF_50%_ of 45% predicted with current spirometry and switched to the group selection with SAD.

Patients were asked about their symptoms, age of asthma onset, possibly related illnesses or allergies and worsening factors or situations such as exercise, weather conditions, psychological stress, and physical fatigue. After 10 in-depth interviews, saturation of topics was attained.

Subsequently, 6 patients with and 5 patients without SAD were identified to attend the focus group interviews. Patients were selected based on a combination of spirometry and IOS measures (Figure 1). Presence of SAD was defined as both FEF_50%_< LLN *and* R5-R20 > 0.10 kPa/L/s. Absence of SAD was defined as both FEF_50%_ > LLN *and* R5-R20 ≤ 0.10 kPa/L/s. The cut-off value for R5-R20 was based on a study population of 110 healthy, never or currently smoking subjects, age 18–73 years (NCT00848406; [19]). Of these subjects, 90% had an R5-R20< 0.10 kPa/L/s.

#### Methods of interviews and focus groups

All useful topics (items) were selected to be further discussed during the focus group interviews. Discussions in the focus groups were literally and fully transcribed by two authors of this article (LSG and EvdW). Qualitative data management software (NVivo 9 [20]) was used. All items mentioned in the in-depth and focus group interviews were organized in groups of items of interest. Then, a list of all items that differed between SAD and non-SAD patients of the focus groups was created. Afterwards, the list was back- and forward translated by a native English speaking pulmonology expert and two Dutch bilingual primary care researchers with knowledge of pulmonology.

The study and procedures were approved by the medical ethics committee of the University Medical Center Groningen and all patients gave written informed consent.

## Results

The clinical characteristics of the 7 asthma patients with SAD and 3 patients without SAD participating in the individual in-depth interviews are presented in Table 2. Both groups of patients were comparable for most clinical parameters, except FEV_1_/FVC ratio and residual volume (RV). A total of 6 asthma patients with and 5 without SAD participated in the focus groups (Table 2). No differences between the group with and without SAD were observed in most parameters, except for lung function parameters. Four patients of the SAD focus group had also participated in the individual in-depth interviews. Patients participating in the in-depth interview or focus group did not use small particle inhalation medication.

**Table 2:** **Patient characteristics participating in the focus groups**

	**In-depth interview group**					**Focus group**				
	**With SAD (n= 7)**		**Without SAD (n= 3)**			**With SAD (n= 6)**		**Without SAD (n= 5)**		
	**median**	**(range)**	**median**	**(range)**	**p-value**	**median**	**(range)**	**median**	**(range)**	**p-value**
Age (years)	45	(25–73)	52	(20–63)	0.833	54	(25–73)	47	(36–51)	0.247
Gender (female/male)	5/2		2/1		1.000	5/1		4/1		1.000
BMI (kg/m^2^)	28	(23–39)	36	(32–38)	0.117	29	(23–52)	32	(24–38)	0.792
Smoking (current/ex/never%)	1/4/2		1/1/1		0.728	0/5/1		0/1/4		0.080
ICS (yes/no)	5/2		3/0		1.000	4/2		3/2		0.497
FEV_1_ (%predicted)	79	(66–104)	99	(94–123)	0.117	81	(76–88)	112	(97–127)	0.004
FEV_1_/FVC (%)	62	(52–73)	83	(81–89)	0.017	64	(52–77)	83	(80–88)	0.004
FEF_50%_ (%predicted)	37	(23–62)	107	(89–115)	NA	40	(23–55)	114	(82–122)	NA
RV (%predicted)	120	(88–160)	72	(71–89)	0.033	120	(88–160)	88	(77–98)	0.017
IOS R20 (kPa/l/s)	0.39	(0.29-0.50)	0.33	(0.32-0.40)	0.517	0.39	(0.34-0.67)	0.38	(0.26-0.49)	0.429
IOS R5-R20 (kPa/l/s)	0.27	(0.10-0.44)	0.19	(0.11-0.25)	0.383	0.25	(0.14-0.29)	0.05	(0.01-0.08)	NA
IOS X5 (kPa/l/s)	−0.22	(−0.49;-0.12)	−0.23	(−0.26;-0.11)	1.000	−0.23	(−0.49;-0.22)	−0.07	(−0.12;-0.06)	0.004
PC_20_ methacholine^#^ (mg/ml)	1.1	(0.1-6.4)	3.5	(1.3-23.9)	0.183	1.13	(0.14-5.55)	9.8	(2.8-20.9^@^)	0.017
ACQ (total score)	0.5	(0.0-1.5)	2.0	(0.0-2.2)	0.383	0.6	(0.0-1.3)	1.0	(0.3-2.2)	0.177
BHQ symptoms (score)	0.4	(0.0-2.5)	1.8	(0.3-3.1)	0.383	1.3	(0.0-2.7)	1.9	(1.3-2.7)	0.329
BHQ stimuli (score)	1.2	(0.0-4.3)	1.8	(0.0-4.3)	0.833	2.5	(0.1-3.8)	2.5	(0.9-4.1)	0.662

^@^Patient with a PC_20_ 20.9 mg/ml methacholine used 800 μg ICS. ^#^Values were log transformed. Differences were tested with a non-parametric test. For ordinal variables differences were tested with the Fisher’s Exact test or Chi-square test as appropriate.

BMI= body mass index, ICS= inhaled corticosteroids, FEV_1_= Forced expiratory flow in one second, FVC= forced vital capacity, FEF_50%_= forced expiratory flow at 50% of the FVC, RV= residual volume, IOS= impulse oscillometry, R20: Resistance of the respiratory system at 20 Hertz, R5-R20: Difference between the resistance at 5Hz and 20Hz, X5= Reactance of the respiratory system at 5 Hertz, PC_20_= provocative concentration causing a 20% fall in FEV_1_, ACQ= Asthma Control Questionnaire, BHQ= Bronchial Hyperresponsiveness Questionnaire.

### Item organization and selection

All items that appeared to be different between the two groups were selected and a total of 63 items was retained. All items were phrased in a positive way (for example “I have an immediate reaction to cats”). Of these phrases, 21 phrases were in line with symptoms of patients with SAD and not of patients without SAD (e.g. “When I feel asthmatic, I feel it in my chest.”), whereas 41 phrases were in line with symptoms of patients without SAD (e.g. “I frequently have a hoarse or husky voice”). In addition one open question (“At what age did you first suffer from asthma symptoms?”) was added. The resulting 63 items are shown in Table 3 and are divided in the following 10 domains.

**Table 3:** **63-items of the SADT**

	**Without SAD**	**With SAD**
*Domain 1: Asthma symptoms*	Concentrating on my breathing helps me when I feel asthmatic.	I’m not able to breathe in deeply when I feel asthmatic.
	People often tell me they can hear me breathing, even in a calm situation.	I sometimes wheeze when I’m at ease or in rest.
	I only wheeze when I feel very asthmatic.	When I’m physically active (like walking the stairs), I sometimes wheeze.
	I often cough unexpectedly.	I can feel suddenly asthmatic without having any other symptoms.
	I often cough superficially (tickling cough) before I get bothered by coughing more deeply.	
	I can see it coming when I get my asthma.	I almost always feel slightly asthmatic and I take a rescue puff regularly.
	I often have a period without feeling asthmatic and without needing rescue puffs.	I have suffered from bronchitis.
*Domain 2: Ear/nose/ throat complaints*	My asthma symptoms are preceded by the flu or a cold.	I usually have runny or painful eyes when I have hay fever.
	When I feel asthmatic, I almost always have symptoms comparable to a cold.	
	I usually get a cold first, and thereafter start coughing.	
	I often suffer with my ears.	
	I often have runny or painful eyes without having hay fever.	
	I frequently have a hoarse or husky voice.	
	When I feel asthmatic, it often comes with symptoms of my throat, nose, ears or eyes.	
	When I feel asthmatic, I often, also suffer from a sore throat.	
	My tonsils or adenoids have been removed.	
*Domain 3: Localization of symptoms*	When I feel asthmatic, I feel it in the middle of my back.	When I feel asthmatic, I have a pressing and oppressive feeling.
	When I feel asthmatic, I feel it in the top of my back.	When I feel asthmatic, I feel it in my chest.
	When I feel asthmatic, I feel a stab or a sting in my back or my ribs.	
	When I feel asthmatic, I sometimes feel bloated.	
*Domain 4: BHR to exercise*	As a child, I always participated in all games and sports.	Actually, I cannot perform strenuous exercise or sport, because I will become asthmatic.
	I am able to walk a long distance without resting.	Physical activities always make my asthma worse.
	When I’m not ill, I can easily do physical activities such as walking the stairs.	
	When I feel asthmatic when exercising, it is very often due to the environment (grass, trees, flowers…).	
	When I feel asthmatic when exercising, it is very often due to the weather.	
	Sometimes I go running or jogging.	
*Domain 5: BHR to allergens*	I cannot stand woolen blankets or clothes.	I have an immediate reaction to birds.
	I cannot stand the down filling in pillows.	I have an immediate reaction to cats.
*Domain 6: BHR to weather changes*	I tire more rapidly due to weather changes.	My asthma worsens in autumn.
	My breathing becomes easier in cold air.	I feel asthmatic more rapidly due to weather changes.
	I always sleep with an open window, otherwise I feel asthmatic.	I feel asthmatic when I suddenly enter a cold environment.
*Domain 7: Stress and fatigue*	I rapidly get tired due to my asthma symptoms.	In stressful situations, I get particularly asthma symptoms.
	Feeling tired is as much part of my asthma as feeling short of breath.	In stressful situations I have physical symptoms such as complaints of the nose, throat or voice.
	I tire more rapidly due to my asthma symptoms.	
	When I’m feeling tired I will probably get asthmatic in a few days.	
	When I feel asthmatic, I often, also have a headache.	
*Domain 8: Gastro-intestinal tract*	Sometimes I feel asthmatic or out of breath because of heartburn.	
	Sometimes, when I’m short of breath, it can be a relief to burp.	
	Sometimes I have stomach problems which can make me feel asthmatic.	
*Domain 9: Skin*		I get eczema because of weather changes.
		I get skin problems (like eczema) when touching some kinds of food (e.g. fruit or vegetables).
		My asthma symptoms and eczema alternate.
*Domain 10: Miscellaneous*	I often get car sick or travel sick.	
	I have more than three close relatives suffering from asthma or comparable illnesses.	
	At what age did you first suffer from asthma symptoms?

1. The *Asthma Symptoms* domain (13 items); patients with SAD reported to wheeze more often and more easily. All patients with SAD were unable to breathe in deeply when having asthma symptoms, while this was not reported by any of the patients without SAD.
2. The *Ears, Nose and Throat* domain (10 items); these symptoms were only mentioned by patients without SAD.
3. The *Localization of Symptoms* domain (6 items) includes items concerning the exact spot of pain and other signs when feeling asthmatic. SAD patients for example mentioned an oppressive feeling and pain in the chest, whereas patients without SAD felt bloated and sometimes had pain in the middle or the upper part of the back.
4. The *BHR to Exercise* domain (8 items); patients with SAD mentioned problems with regular physical activities, while those without SAD were rarely hindered in their activities.
5. The *BHR to Allergens* domain (4 items) shows that patients with SAD react to birds and cats, while those without SAD cannot stand wool or down.
6. The *BHR to Weather Changes* domain (6 items) reflects that both groups seem to react to weather changes, but in a different way.
7. The *Stress and Fatigue* domain (7 items); patients with SAD reported more asthma symptoms in relation to stress, while patients without SAD reported more asthma symptoms related to periods of fatigue.
8. The *Gastrointestinal Complaints* domain (3 items); Patients without SAD mentioned to suffer sometimes from gastrointestinal problems related to their asthma, but asthma patients with SAD did not.
9. The *Skin problems* domain (3 items); only patients with SAD reported eczema related to asthma.
10. The last domain (3 items) was named *Miscellaneous*. Patients without SAD reported often getting car-sick and having asthmatic relatives. Patients with SAD reported to be somewhat older when their first symptoms appeared. These items did not combine with any other domains and were thus placed in this last domain.

## Discussion

This is the first phase of the development of a questionnaire aiming to help identifying asthma patients with SAD based on self-reported symptoms in clinical practice. Based on the differences that we found after ten in-depth interviews and two focus groups in patients with and without SAD, we identified a total of 63 items that may help to differentiate between patients with and without SAD. In short, we found that patients with SAD reported to wheeze easily, were unable to breathe in deeply, mentioned more symptoms related to BHR, experienced more pronounced exercise-induced symptoms and more frequently had allergic respiratory symptoms after exposure to cats and birds.

Interestingly, a number of the observed differences between patients with and without SAD, are supported by recent observations reported in the literature [21-31]. Our finding that patients with SAD were unable to breathe in deeply and reported to wheeze very easily may reflect hyperinflation, compatible with small airway closure. Mansur et al. also found a relationship between more symptoms of wheezing and more severe small airways dysfunction during a methacholine provocation test [21]. In addition, patients with SAD indicated that they had suffered from bronchitis at least once in their life, whereas patients without SAD did not. More frequent symptoms and need of rescue treatment are compatible with worse asthma control and the occurrence of asthma exacerbations, findings that have been related to SAD in asthma in previous studies as well [22-24].

Patients with SAD also reported more frequently symptoms of BHR to exercise, allergens and weather changes (domains 4, 5 and 6). This is compatible with earlier studies on BHR showing that hyperresponsiveness is associated with small airways obstruction in asthma [25-27]. Interestingly, Zeidler et al. showed that patients with allergic asthma exposed to cat allergens have predominantly a response of the small airways measured by HRCT scan [28]. Our asthma patients with SAD reported an immediate and strong response to cats. Indeed, these allergens can be found on rather small particles (diameter<2.5 μm), probably affecting asthma patients with SAD more than patients without SAD [29]. In contrast, patients without SAD reported to respond strongly to wool and down. This might be related to house dust-mite excretion in these tissues, which is found on larger particles (10 μm) [30]. Exercise induced symptoms were predominantly mentioned by patients with SAD, which is in line with a study of Lee et al. showing an association between the severity of exercise-induced response and an increased resistance of the small airways [31].

For some of the observed differences between patients with and without SAD we have not found corresponding findings in the existing literature. These novel findings might be interesting to study in a more systematic way in further research regarding symptoms of SAD in asthma.

Our patients represent the asthma population as present in the original primary care database of the asthma COPD service from “Certe Laboratory”. Included patients had a median age of 51 years and a median BMI of 30 kg/m^2^, 77% of the patients being female, whereas the asthma-population of the primary care database had a mean age of 53 with a mean BMI of 29 kg/m^2^ and consisted of 68% females. The smoking habits of our study population, i.e. 12/47/41% current/never/ex smokers, were comparable with the asthma-population of the primary care database. Thus the final list of 63 items is not limited to non-smokers. This is of importance since a few studies have suggested an important effect of smoking on SAD [32,33]. With exclusion of smokers we could have missed signs and symptoms important for SAD.

The study has some limitations. The scientific community has so far not provided an agreed gold standard for the diagnosis of SAD in asthma. We did not use Computed Tomography (CT scan) or multiple breath nitrogen washout tests to assess the presence of small airways disease, yet we used a very precise way of selecting and reselecting patients with and without SAD, based on a combination of both FEF_50%_ and R5-R20 values. These parameters are frequently described as a reliable way to assess SAD [8,10]. The division in patients below and above the LLN of the FEF_50%_ has also been used in a previous study to compare patients with and without SAD [26]. For the R5-R20 we used a cutoff value that was based on a group of 110 well selected and well characterized healthy controls [19].

In summary, the present study is a first step in the development of the SADT. We generated 63 items for the new small airways dysfunction tool, the SADT, which aims to identify patients with SAD. The items that were identified cover a broad area of asthma symptoms related to airway hyperresponsiveness, response to allergens and physical exercise. All generated items will be further tested and validated in a large asthma population with a wide spectrum of severity during a multinational longitudinal study that will start in the near future. The next study will retain the most relevant items with which a short and simple tool to determine SAD in asthma patients will be realized.

### Clinical implications

Identification of SAD in asthma patients may be possible using this simple and non-invasive tool. Differences in symptoms and signs between asthma patients with and without SAD were identified.

### Capsule summary

Based on differences in symptoms and signs, habits and health related issues between asthma patients with and without SAD, 63 items were combined to be entered in the next phase of the SADT.

## Additional file

Additional file 1
**Measurement specifications.**

## Abbreviations

ACQ: Asthma Control Questionnaire
ATS: American Thoracic Society
AX: Reactance area
BHR: Bronchial hyperresponsiveness
BHQ: Bronchial Hyperresponsiveness Questionnaire
BMI: Body mass index
CCQ: Clinical COPD Questionnaire
CT scan: Computed tomography scan
FEF_25–75%_: Forced expiratory flow at 25% to 75% of FVC
FEF_50%_: Forced expiratory flow at 50% of FVC
FEV_1_: Forced expiratory flow in one second
FVC: Forced vital capacity
IOS: Impulse oscillometry
IQ: Interquartile
LLN: Lower limit of normal
NCT: Clinical trial registry number
PC_20_: Provocative concentration causing a 20% fall in FEV_1_
X5: Reactance of the respiratory system at 5 Hertz
R5: Resistance of the respiratory system at 5 Hertz
R20: Resistance of the respiratory system at 20 Hertz
R5-R20: Difference of R5 minus R20
RV: Residual volume
SAD: Small airways dysfunction
SADT: Small Airways Dysfunction Tool
