# Supplementary material for: Development of a tool to recognize small airways dysfunction in asthma (SADT)
Source: Health Qual Life Outcomes. 2014 Nov 22;12:155. doi: 10.1186/s12955-014-0155-7 (PMC4253607; doi:10.1186/s12955-014-0155-7)
Supplement: Additional file 1 — Measurement specifications. [file 12955_2014_155_MOESM1_ESM.docx]

**Measurement specifications**

Measurements

*Questionnaires*

The patient questionnaires included the Dutch versions of the Asthma Control Questionnaire (ACQ), the Clinical COPD Questionnaire (CCQ) and the Bronchial Hyperresponsiveness Questionnaire (BHQ). The ACQ with 6 questions was used, including 5 questions about symptoms and one question about the use of short-acting beta2-agonists(1). The CCQ is a short health status questionnaire for patients with COPD aimed at obtaining information on the severity of symptoms and limitations and can add valuable information to the ACQ in patients with asthma(2). The BHQ is constructed as a condition-specific instrument for measuring the presence and severity of BHR in patients with asthma(3). The BHQ consists of 2 domains, i.e. 15 questions on asthma symptoms and 19 on stimuli that can trigger asthma.

*Lung function*

Spirometry and body plethysmography (Masterlab, Viasys Healthcare) were performed according to the international guidelines(4,5). A daily calibrated pneumotachograph was used throughout this study. Predicted values were obtained from Quanjer et al(6). IOS measurements have been performed (IOS masterscreen, E. Jaeger, Wurzburg, Germany) according to standard recommendations(7). Parameters assessed with this test included resistance at 5 and 20 Hz (R5, R20, R5-R20), reactance at 5Hz (X5) and reactance area (AX).

*Methacholine provocation test*

The bronchial provocation test was performed with the Jaeger APS Pro system with the Medic-Aid sidestream nebulizer (Viasys Healthcare, Höchberg, Germany) powered by compressed air. The provocation test was performed with methacholine bromide (0.038–39.3 mg/ml) according to the tidal breathing method as described in the ERS guidelines, with a small modification(8). An IOS measurement was performed after each inhalation step at 30s and an FEV_1_ value measurement at 90s. The provocative concentration causing a 20% fall in FEV_1_ (PC_20_) was calculated by log-linear interpolation.

References

(1) Juniper EF, O'Byrne PM, Guyatt GH, Ferrie PJ, King DR. Development and validation of a questionnaire to measure asthma control. Eur Respir J 1999 Oct;14(4):902-907.

(2) van der Molen T, Willemse BW, Schokker S, ten Hacken NH, Postma DS, Juniper EF. Development, validity and responsiveness of the Clinical COPD Questionnaire. Health Qual Life Outcomes 2003 Apr 28;1:13.

(3) Riemersma R, Postma D, Kerstjens H, Buijssen K, Boezen M, Aalbers R, et al. Development of a questionnaire for the assessment of bronchial hyperresponsiveness. Prim Care Respir J 2009 12;18(4):287-293.

(4) Miller MR, Hankinson J, Brusasco V, Burgos F, Casaburi R, Coates A, et al. Standardisation of spirometry. Eur Respir J 2005 08;26(2):319-338.

(5) Wanger J, Clausen JL, Coates A, Pedersen OF, Brusasco V, Burgos F, et al. Standardisation of the measurement of lung volumes. Eur Respir J 2005 09;26(3):511-522.

(6) Quanjer PH, Tammeling GJ, Cotes JE, Pedersen OF, Peslin R, Yernault JC. Lung volumes and forced ventilatory flows. Report Working Party Standardization of Lung Function Tests, European Community for Steel and Coal. Official Statement of the European Respiratory Society. Eur Respir J Suppl 1993 03;16:5-40.:5-40.

(7) Oostveen E, MacLeod D, Lorino H, Farre R, Hantos Z, Desager K, et al. The forced oscillation technique in clinical practice: methodology, recommendations and future developments. Eur Respir J 2003 12;22(6):1026-1041.

(8) Sterk PJ, Fabbri LM, Quanjer PH, Cockcroft DW, O'Byrne PM, Anderson SD, et al. Airway responsiveness. Standardized challenge testing with pharmacological, physical and sensitizing stimuli in adults. Report Working Party Standardization of Lung Function Tests, European Community for Steel and Coal. Official Statement of the European Respiratory Society. Eur Respir J Suppl 1993 03;16:53-83.:53-83.
